# False-Negative Ligamentum Teres Tendon Sampling for the Detection of Systemic Amyloidosis

**DOI:** 10.7759/cureus.116430

**Published:** 2026-09-17

**Authors:** Thomas R Yetter, Zachary D Mendoza, Roxanne R Miller, Barry H Trachtenberg, Shari R Liberman

**Affiliations:** 1 Department of Orthopedics and Sports Medicine, Houston Methodist Hospital, Houston, USA; 2 School of Engineering Medicine, Texas A&M University, Houston, USA; 3 DeBakey Heart and Vascular Center, Houston Methodist Hospital, Houston, USA

**Keywords:** amyloidosis, biopsy, carpal tunnel syndrome, hip arthroplasty, lumbar spinal stenosis

## Abstract

This case report involves a 60-year-old man with bilateral carpal tunnel syndrome, hip osteoarthritis, and lumbar spinal stenosis who underwent biopsies of the ligamentum teres, ligamentum flavum, and carpal flexor tenosynovium. Amyloid deposits were identified in the ligamentum flavum and flexor tenosynovium, but not the ligamentum teres. Amyloid was typed by mass spectrometry, cardiac scintigraphy was negative, and genetic testing remains pending. This supports that ligamentum flavum and carpal flexor tenosynovium biopsies may have better diagnostic value for the early diagnosis of amyloidosis, while the ligamentum teres is an unpredictable location of biopsy for early diagnosis.

## Introduction

Amyloidosis is the extracellular deposition of insoluble protein or protein fragments throughout the body that results in organ dysfunction [1,2]. The incidence of amyloidosis is reported from 9.7 to 14 cases per million people per year; however, it is believed that amyloidosis is underdiagnosed due to nonspecific clinical presentations and overlapping medical conditions [2,3]. Amyloidosis can have clinical manifestations of the cardiac, gastrointestinal, renal, hepatic, nervous, and musculoskeletal systems [1]. Specifically, wild-type transthyretin amyloidosis (wtATTR) is the most common form of amyloidosis presenting with cardiac and orthopedic manifestations. Transthyretin amyloidosis (ATTR) results from the deposition of misfolded transthyretin and may be hereditary, due to the pathogenic variants in the transthyretin (TTR) gene, or wild-type (wtATTR), occurring without an identifiable TTR mutation. Cardiac amyloidosis refers specifically to amyloid deposition within the myocardium and may result from ATTR or other amyloid proteins, most notably immunoglobulin light-chain amyloid. In wtATTR, amyloid deposition may occur in musculoskeletal tissues years before clinically apparent cardiac involvement [1-3]. Various treatments for amyloidosis, including both surgical and pharmacologic therapies, have been shown to be effective [4]. However, with more advanced disease at the time of diagnosis, patients are left with fewer treatment options and decreased life expectancy [4].

Traditionally, diagnosis of wtATTR required a tissue biopsy historically taken from the abdominal fat pad, bone marrow, salivary gland, rectum, or affected organ; however, biopsy is not always necessary for definitive diagnosis [1]. The tissue is then stained with Congo red dye, which detects amyloid deposits that show apple-green birefringence under polarized light microscopy [1,5]. Once the presence of amyloid is confirmed, amyloid typing is performed to identify the specific protein present [5]. Knowing this, some have proposed the use of different tissue biopsies in patients with earlier disease manifestations to detect wtATTR sooner, begin treatment earlier, and prevent life-threatening manifestations like cardiac amyloidosis.

There is a growing amount of evidence and utility in the utilization of musculoskeletal biopsies in the setting of routine orthopedic procedures to aid in the diagnosis of amyloidosis. It has been shown that bilateral carpal tunnel syndrome (CTS), lumbar spinal stenosis (LSS), and biceps tendon rupture may precede symptoms of cardiac amyloidosis by years [1-3,6]. The association of bilateral CTS and LSS with amyloidosis has led to the evaluation of the carpal flexor tenosynovium, transverse carpal ligament (TCL), and ligamentum flavum as intraoperative biopsy sites for the earlier diagnosis of wtATTR [7,8]. Biopsies of these sites are considered reliable, safe, convenient, inexpensive, and available at most institutions [7,8]. Carpal tunnel tenosynovium biopsies during carpal tunnel release have emerged as a routine practice for many surgeons due to their reliability in the diagnosis of wtATTR [7]. Similarly, ligamentum flavum biopsies during surgery in patients with LSS have been shown to be amyloid positive in over one-third of patients [9]. Additionally, it has been shown that compared to the general population, patients with cardiac amyloidosis had significantly higher rates of both total hip and knee arthroplasty [10]. However, the comprehensive spectrum of musculoskeletal tissues that may harbor amyloid deposits, and their relative diagnostic utility, remains an area of active investigation [11].

Given the growing evidence of musculoskeletal manifestations as early indicators of wtATTR, intraoperative biopsies during routine orthopedic procedures are becoming common practice for many surgeons. To our knowledge, there is no published literature regarding the efficacy of ligamentum teres biopsy during total hip arthroplasty for the early detection of amyloidosis. This case report aims to further explore this landscape by presenting a patient with bilateral CTS, LSS, and hip osteoarthritis, whose diagnostic workup included discordant amyloid detection among three musculoskeletal biopsy sites of the carpal flexor tenosynovium, ligamentum flavum, and ligamentum teres. The ligamentum teres is routinely removed during total hip arthroplasty and is an easily accessible tissue that could potentially aid in the early diagnosis of amyloidosis.

## Case presentation

A 60-year-old man with no pertinent cardiac history presented with a two-year history of bilateral hip pain (day 1). He denied any pertinent family history of amyloidosis. He denied any autonomic symptoms, renal abnormalities, macroglossia, or prior tendon rupture. He was found to have severe bilateral hip degenerative joint disease upon radiographic evaluation (Figure 1A).

**Figure 1:**
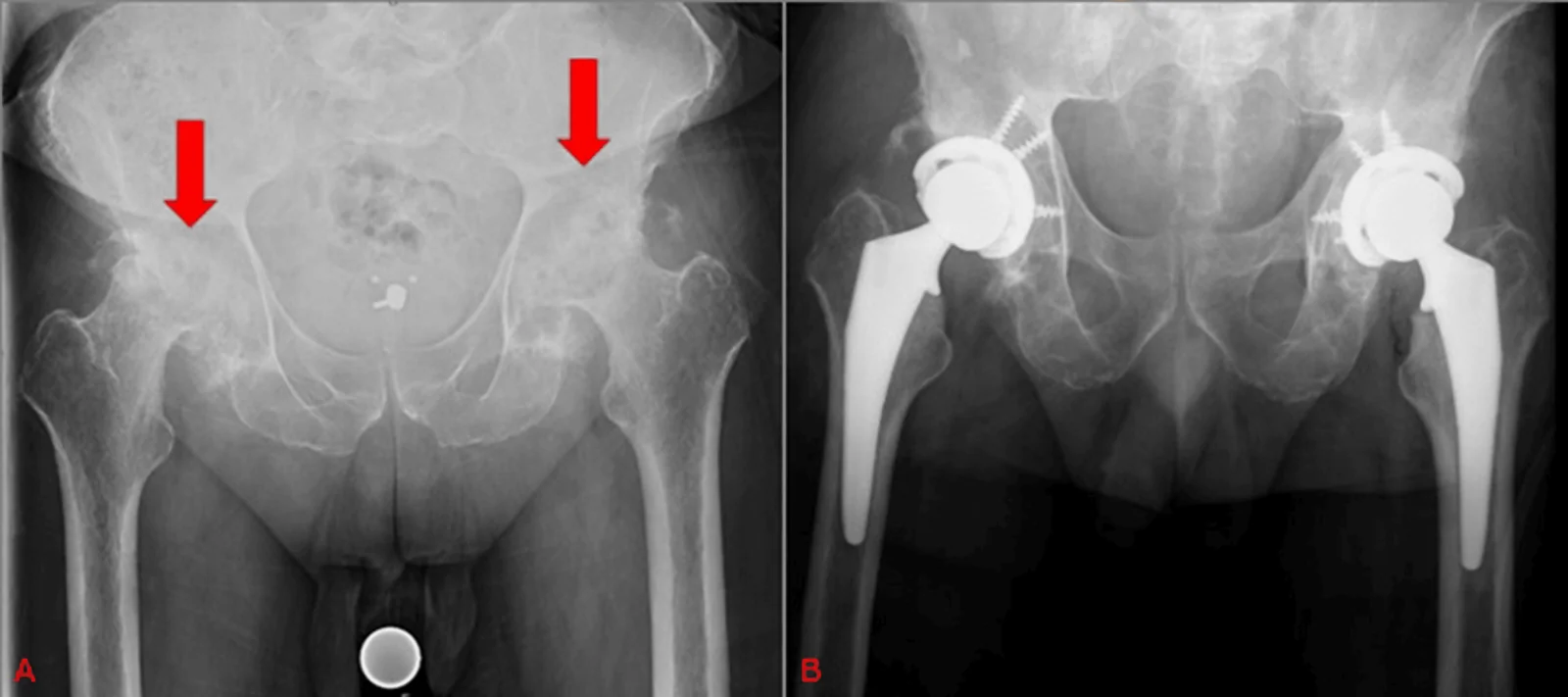
(A) Preoperative AP pelvis radiograph demonstrating severe end-stage osteoarthritis of both hips. (B) Postoperative radiograph following bilateral total hip arthroplasty. AP: anteroposterior

He reported persistent symptoms in bilateral hips, low back, and bilateral hands after trialing non-operative management including anti-inflammatory medications, corticosteroid injections, physical therapy, weight loss, activity modification, and assistive gait aids. Preoperatively, he was an active person; however, he had significantly decreased ambulatory distances limited by his hip and back pain. Over the course of his non-operative management, he began having worsening pain, stiffness, and weakness in bilateral lower extremities. He subsequently underwent right total hip arthroplasty on day 134 and left total hip arthroplasty on day 190 (Figure 1B). Then, the patient was evaluated in the clinic for a four-year history of bilateral hand numbness and weakness refractory to non-operative management including bilateral carpal tunnel corticosteroid injections. He additionally reported significant dysfunction from bilateral ring trigger fingers. Preoperatively, he reported reduced bilateral grip strength and worsening difficulty with manual dexterity. Over the course of his non-operative management, he began having worsening pain, stiffness, and weakness in bilateral hands. Electromyography (EMG) revealed severe bilateral CTS and moderate cubital tunnel syndrome. He subsequently underwent right open carpal tunnel release, cubital tunnel release, and trigger finger release on day 233. Then, he underwent left open carpal tunnel release, cubital tunnel release, and trigger finger release on day 247. He was then evaluated for his chronic low back pain, which persisted despite non-operative management including physical therapy, activity modification, weight loss, and transforaminal corticosteroid injections. Imaging revealed severe LSS from L2 to L5, and the patient underwent L2-L5 lumbar spinal decompression on day 258 (Figure 2).

**Figure 2:**
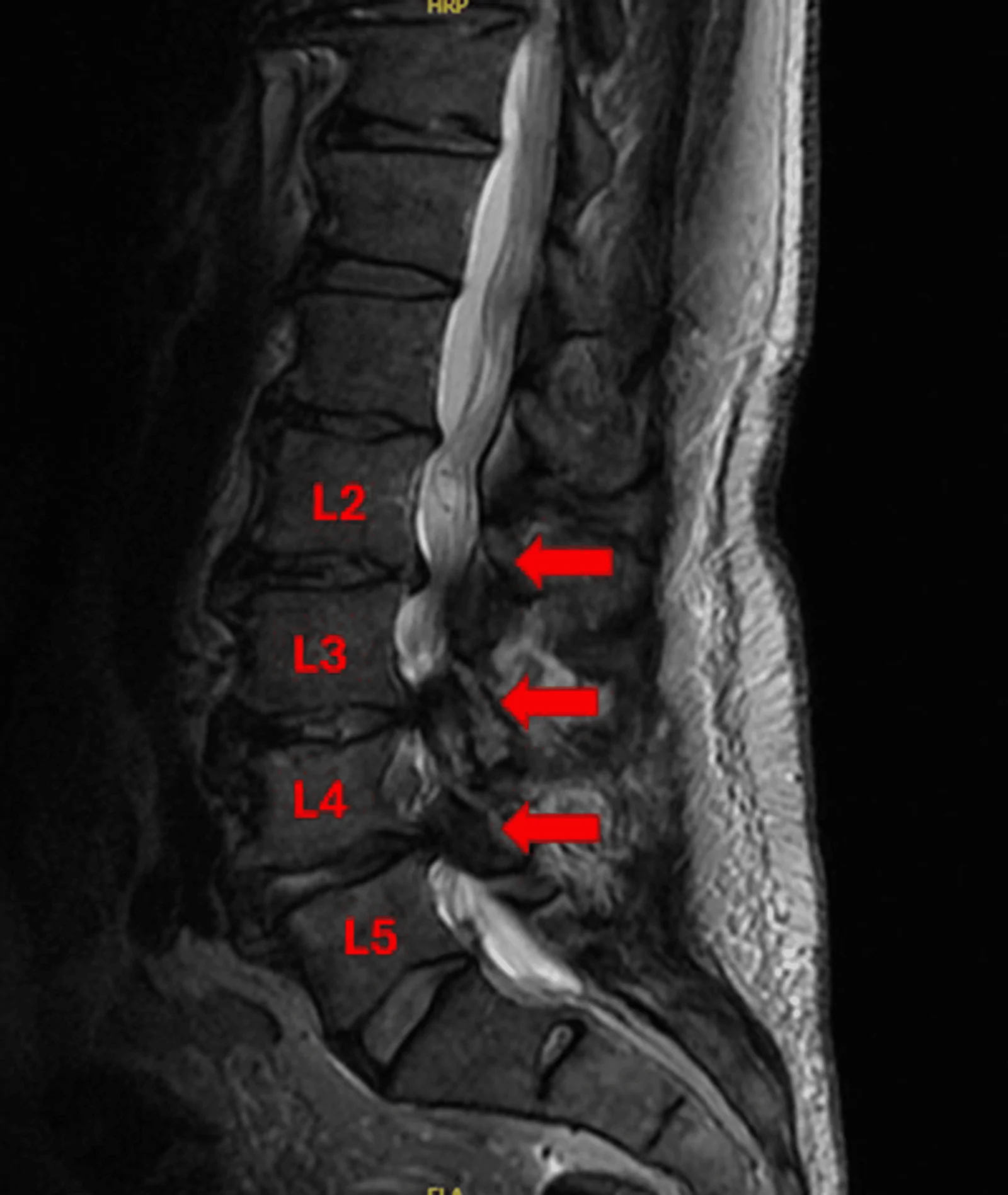
Lumbar spine T2 sagittal MRI demonstrating multilevel degenerative disc disease and spondylotic changes with spinal canal and foraminal stenosis from L2 to L5.

Secondary to his age and multiple musculoskeletal complaints, there was a high index of suspicion for amyloidosis. Thus, biopsies of the ligamentum teres, ligamentum flavum, and carpal flexor tenosynovium were taken at the time of the respective surgeries. Amyloid deposition on Congo red stain was identified within the ligamentum flavum and carpal flexor tenosynovium, but not the ligamentum teres. Histopathological images of biopsies were not readily available in the medical record for inclusion in this manuscript. The biopsies from the spine and wrist were confirmed as wtATTR amyloidosis by liquid chromatography-tandem mass spectrometry (LC-MS/MS). He was subsequently referred to cardiology for evaluation for cardiac amyloidosis (day 331). Laboratory investigation to exclude light-chain amyloidosis was performed by the cardiology team. Evaluation revealed no involvement with technetium-99m pyrophosphate (99mTc-PYP) scintigraphy. He is pending cardiac MRI and ATTR amyloid genetic testing based on cardiology recommendations. He will continue to follow up with cardiology for monitoring for possible future cardiac involvement. Complete pathology characteristics including specimen dimensions, number of tissue blocks and levels examined, Congo red staining protocol, distribution, and approximate burden of amyloid within biopsy tissues were not readily available in the electronic medical record and performed per institutional protocol and surgeon judgment. A timeline of the procedure and biopsy results can be found in Table 1.

**Table 1:** Summary timeline of procedures, biopsies, and amyloid biopsy results.

Day	Surgery	Biopsy site	Amyloid biopsy result
134	Right total hip arthroplasty	-	-
190	Left total hip arthroplasty	Ligamentum teres	Negative
233	Right open carpal tunnel release, cubital tunnel release, and trigger finger release	Carpal flexor tenosynovium	Positive
247	Left open carpal tunnel release, cubital tunnel release, and trigger finger release	-	-
258	L2-L5 lumbar spinal decompression	Ligamentum flavum	Positive

## Discussion

Early diagnosis of ATTR is crucial, as it can prevent life-threatening manifestations and improve patient outcomes [12,13]. ATTR can present with diverse musculoskeletal symptoms including bilateral CTS, LSS, biceps tendon rupture, arthropathies, and neuropathies [1-3,6-11]. Orthopedic surgeons should maintain a high index of suspicion for ATTR in patients over 50 years old who present with multiple musculoskeletal complaints. Tissue biopsies during routine orthopedic surgeries, such as carpal flexor tenosynovium and ligamentum flavum biopsies, can aid in the early diagnosis of ATTR [7-9]. These biopsies are within the standard approach of the surgery and can be easily performed [7-9]. Early detection allows for timely intervention and improves long-term outcomes for patients with ATTR [12,13]. By incorporating tissue biopsy into their clinical practice, orthopedic surgeons can play a significant role in the early diagnosis and management of this disease.

However, the utility of ligamentum teres biopsy in the setting of total hip arthroplasty is not well described in the literature, and to our knowledge, there are no published studies evaluating this. The biopsy of the ligamentum teres in this patient was negative for amyloidosis less than two months prior to his carpal tunnel and lumbar stenosis surgeries. This would suggest that the ligamentum teres may be an unreliable site for biopsy for the early detection of amyloidosis.

However, definitive claims cannot be made regarding this case report as the findings in a single patient can have many causations of sampling error. Inadequate quality or quantity of tissue samples, nonstandardized tissue volumes, absence of diagnostic-accuracy estimates, incomplete genetic evaluation, and limited follow-up are all possible confounding factors that would impact the results in this single patient. Additional investigation via studies with larger sample sizes is necessary to fully understand the efficacy of ligamentum teres biopsy for the detection of amyloidosis.

## Conclusions

We discussed a 60-year-old patient with chronic bilateral hip osteoarthritis, bilateral CTS and cubital tunnel syndrome, and LSS with biopsy-proven amyloidosis. This case supports that ligamentum flavum and carpal flexor tenosynovium biopsies aid in the early detection of amyloidosis. However, it questions the utility of ligamentum teres biopsies for the early detection of amyloidosis. There is limited literature evaluating the efficacy of ligamentum teres biopsies for amyloidosis, and this is an area for future investigation.
